# Diagnosis, treatment, and genetic characteristics of blastic plasmacytoid dendritic cell neoplasm: A review

**DOI:** 10.1097/MD.0000000000032904

**Published:** 2023-02-17

**Authors:** Yemin Wang, Li Xiao, Lili Yin, Lv Zhou, Yanjuan Deng, Huan Deng

**Affiliations:** a Department of Pathology, Fourth Affiliated Hospital of Nanchang University, Nanchang, Jiangxi, China; b Mol. Med. & Genet. Center, Fourth Affiliated Hospital of Nanchang University, Nanchang, Jiangxi, China.

**Keywords:** blastic plasmacytoid dendritic cell neoplasm, chimeric antigen receptor T cells, genetics, tagraxofusp, targeted therapy, venetoclax

## Abstract

Blastic plasmacytoid dendritic cell neoplasm (BPDCN) is a highly aggressive and extremely rare hematologic disease with a poor prognosis, involving mainly the skin and bone marrow. The immunophenotype of these tumor cells is characterized by the expression of CD4, CD56, CD123, TCL-1, and CD303. To date, no consensus has been reached on the standard of care for BPDCN. Currently, clinical treatment is mainly based on high-dose chemotherapy combined with hematopoietic stem cell transplantation. However, this treatment method has limitations for elderly, frail, and relapsed/refractory patients. In recent years, breakthroughs in molecular biology and genetics have not only provided new ideas for the diagnosis of BPDCN but also helped develop targeted treatment strategies for this disease. The emergence of targeted drugs has filled the gap left by traditional therapies and shown great clinical promise. This article focuses on the latest advances in genetics and targeted therapies for BPDCN, especially the emerging therapies that may provide new ideas for the clinical treatment of BPDCN.

## 1. Introduction

Blastic plasmacytoid dendritic cell neoplasm (BPDCN) is a rare hematological malignancy originating from precursors of plasmacytoid dendritic cells. The nomenclature of this disease had not been standardized in the past due to its unknown origin. However, in 2008, the World Health Organization described BPDCN as a type of acute myeloid leukemia (AML), and in the 2016 revision, it was classified as its own entity.^[1,2]^ BPDCN is characterized by the expression of CD4, CD56, and the pDC-specific associated antigens CD123, TCL-1, and CD303 on its cell surface. Of these, overexpression of CD123 is present in almost all cases of BPDCN.^[3,4]^ Furthermore, genetic abnormalities are common in BPDCN, however, they are heterogeneous, and their exact mechanisms of occurrence and prognostic implications remain controversial. Due to its rare and highly invasive nature, the development of standard care for BPDCN has been challenging. Chemotherapy combined with hematopoietic stem cell transplantation (HSCT) is the traditional treatment for BPDCN and can effectively improve the overall survival of patients.^[5–7]^ However, the use of this method is limited in elderly patients with poor systemic status and relapsed/refractory (R/R) patients. Although drugs targeting CD123, especially tagraxofusp, are attractive candidates, they are far from sufficient.^[8,9]^ Therefore, the biology and pathogenesis of BPDCN should be further explored from a genetic perspective to develop more effective therapeutic strategies and improve the prognosis of BPDCN.

In this study, we aimed to review the research progress on the biological characteristics, gene mutations, and molecular mechanisms of BPDCN. We also summarized the latest research results on the treatment of BPDCN, focusing on targeted therapy, which may be clinically significant in the future.

## 2. Methods

We searched online databases, including Google Scholar, PubMed, and Web of Science for articles from the time of database creation to October 8, 2022, regardless of language, article type, and other restrictions. We used the search terms, blastic plasmacytoid dendritic cell neoplasm, tagraxofusp, genetics, and targeted therapies. Literature related to the diagnosis, treatment, and genetics of BPDCN was included, and gray literature was excluded. After a comprehensive and systematic evaluation of the relevant literature, a total of 76 articles were included.

## 3. Clinical presentation

BPDCN usually affect the skin, and the blood lines, but are heterogenous. Elderly males tend to have a higher risk of BPDCN, with a median age of 53 years and a male to female ratio of 3:1.^[10]^ Skin lesions are characteristic of the disease, and approximately 90% of cases begin with 1 or more persistent skin lesions that may vary in size (from mm to cm), color (from tan to purplish red), and morphology (from nodules to plaques).^[11,12]^ Most patients begin with localized lesions that gradually spread to the whole body, while few patients have only leukemia-like symptoms and no skin lesions.^[3,13,14]^ As the disease progresses, the bone marrow and peripheral blood become involved in 45% of patients,^[12]^ resulting in enlargement of the liver, spleen, and lymph nodes and a reduction in blood cells. Additionally, central nervous system infiltration occurs in about 1-third of patients, necessitating routine cerebrospinal fluid screening by flow cytometry.^[3,15]^ In addition, very rarely, BPDCN may involve the gallbladder, lungs, breasts, kidneys, and bladder.^[16–19]^ In conclusion, the clinical manifestations of BPDCN are varied, and the specificity is weak, resulting in missing or misdiagnosing the disease.

## 4. Morphology and immunophenotype

Morphologically, BPDCN consists of monomorphic and medium-sized blast cells. The nucleus is ovoid or irregular and is often located at the periphery, and the chromatin is fine and scattered. Additionally, small nucleoli may be observed, and the cytoplasm is sparse and lacks granules.^[19]^ Moreover, skin biopsy has shown that malignant cells are diffusely distributed in the dermis and subcutaneous tissue rather than the epidermis, without vascular invasion or necrosis.^[2,20]^ Finally, blast cells on bone marrow puncture smears display a “pearl necklace” appearance with tiny vacuoles extending in a pseudopod-like pattern along the cytoplasmic membrane.^[21]^

The definitive diagnosis of BPDCN can be based on the immunohistochemical results, which are characterized by the expression of CD4, CD56, and the pDC-specific markers CD123, TCL-1, and BDCA-2/CD303 and lack of lymphoid and myeloid markers. Some studies have indicated that in addition to the typical markers mentioned above, positivity for CD33, CD43, CD68, CD79a, CD2, CD7, TDT, HLA-DR, BCL-2, and S-100 is also observed in BPDCN.^[20,22,23]^ In fact, only 46% of patients express the 5 most characteristic markers of BPDCN simultaneously.^[23]^ Moreover, some cases are negative for typical markers and positive for atypical markers, making the diagnosis more challenging. This requires professionals to fully integrate clinical manifestations, morphological features, and immunophenotypes in the diagnosis of BPDCN. Experts now agree that a reliable diagnosis can be made when 4 of the 5 typical markers are expressed.^[4]^

## 5. Cytogenetic aberrations

Patients with BPDCN often harbor cytogenetic abnormalities, and up to 75% of patients exhibit a complex karyotype.^[24]^ Moreover, deletions are more common than amplifications, but the absence of any chromosomal alterations is considered a unique genetic manifestation of the disease. Common chromosomal deletions have been identified in previous studies, mainly at 5q21 or 5q34, 12p13, 13q13-q21, 6q23-qTER, 15q, and throughout chromosome 9.^[25]^ Notably, duplicated chromosomal deletions in BPDCN often result in gene loss at related loci, mainly including oncogenic factors (TP53, PTEN, NR3C1), cell cycle regulators (CDKN1B, CDKN2A/CDKN2B), and transcription factors (ETV6, IKZF1).^[26–29]^ Among them, 9p21.3 (CDKN2A/CDKN2B) deletion, which is associated with poor prognosis, and 12p/ETV6 deletion, which may represent early clonal events, are common in BPDCN.^[27,28]^

Approximately 40% of patients with BPDCN exhibit recurrent rearrangements of MYC (located in 8q24), which is the key regulator of cell growth and proliferation.^[30]^ Sakamoto et al^[30]^ found that MYC^+^BPDCN and MYC^-^BPDCN were different not only in cytological morphology, immunophenotype, and clinical manifestations, but also in sensitivity to bromodomain and extra-terminal protein inhibitors (BETis) and aurora kinase inhibitors, which have potential application value in hematological malignancies.^[31,32]^ BETis can specifically inhibit the MYC-dependent transcription network, thus limiting cell proliferation and differentiation.^[33]^ Moreover, BETis have been shown to have favorable in vitro and in vivo effects on the BPDCN cell line CAL-1.^[29,34]^ Meanwhile, aurora kinase inhibitors can initiate rapid apoptosis and delay autophagy, selectively killing overexpressed MYC tumor cells; this lethal interaction has been verified in human tumor cell lines and mouse tumor models.^[35]^ In conclusion, the above studies suggest that MYC status may be a potential biomarker for the treatment of BPDCN with these inhibitors.

Recently, Sumarriva et al^[36]^ found that t (6;8) (p21;q24) is the most common type of MYC rearrangement, which is related to the increase in the age of onset and the decrease in the median survival time. Later, Kubota et al^[37]^ proved that due to t (6;8)(p21;q24), the pDC-specific RUNX2 super enhancer is juxtaposed with the MYC promoter. RUNX2 (located at 6p21) regulates the expression of genes related to pDC differentiation and migration, and enables BPDCN cell survival and proliferation.^[38]^ The pDC-specific RUNX2 super enhancer was hijacked, which not only promotes RUNX2 expression, but also activates MYC transcription, which promotes the occurrence and development of BPDCN.^[37]^ In addition, MYB and MLL rearrangement are also observed in BPDCN, and MYB may be a major driver of BPDCN in children.^[39–41]^

Although cytogenetic abnormalities are relatively common in BPDCN, they are not specific. Therefore, further exploration of the potential role of cytogenetic abnormalities in BPDCN and their association with molecular biology and clinicopathology is warranted.

## 6. Molecular genetic abnormalities

To determine the genealogical origin of BPDCN, Jardin et al^[42]^ performed genetic testing on 13 patients with BPDCN and identified TET2 and TP53 mutations. Menezes et al^[43]^ comprehensively analyzed 25 patients with BPDCN using next-generation sequencing technology, and found that TET2 was the most commonly mutated gene, followed by ASXL1, NRAS/NPM1, IKZF1/2/3, and ZEB2. Mutations in genes involved in the DNA methylation pathway are usually associated with poor prognosis, suggesting that epigenetically modified genes may be a potential target for BPDCN treatment. Stenzinger et al^[26]^ further extended and complemented the mutational map of BPDCN, uncovering mutations in NRAS, ATM, MET, KRAS, IDH2, and KIT, among which NRAS, KRAS, and ATM have mutually exclusive distributions and may represent different subgroups of BPDCN. Moreover, the frequency of RAS mutations in BPDCN is approximately 57.6%, and targeting this signaling pathway may become a promising therapeutic modality for BPDCN.

In addition to mutations involving epigenetic regulators and signal transduction pathways, RNA splicing factor-encoding gene alterations, including ZRSR2, SRSF2, SF3B1, U2AF1, SF3A2, and SF3B4 also appear in BPDCN, resulting in abnormal RNA shearing, blocking the pathway of pDC maturation or activation, and ultimately promoting tumor formation.^[44–46]^ Lee et al^[47]^ discovered that the splicing inhibitor E7107 significantly reduced the leukemic load in syngeneic mouse leukemia and patient-derived xenograft AMLs carrying splicing factor mutations. Consequently, for patients with BPDCN with mutations in genes encoding RNA splicing factors, splicing regulatory drugs targeting these genes may be a potential therapeutic approach.

Another mutant gene, KZF1, which encodes a transcription factor indispensable for pDC precursor differentiation, has also recently attracted attention. Bastidas et al^[48]^ performed a genome-wide analysis and found that this gene is locally inactivated mainly through repetitive structural changes that affect the development and differentiation of plasmacytoid dendritic cells, thus leading to BPDCN transformation. Therapeutic methods aimed at restoring the function of KZF1 may be a valuable option for BPDCN. In summary, mutations in patients with BPDCN mainly affect 6 types of functional genes, including those involved in DNA methylation, histone modification, signal transduction, transcription factors, cell cycle regulators, and RNA splicing factors (Table 1). Moreover, TET2 and ASXL1 are the most frequently mutated genes. Therapeutic approaches targeting these mutated genes, although distantly promising, may be a new way to challenge the traditional treatment paradigm for BPDCN.

**Table 1:** Gene mutation analysis of BPDCN in the literature.

Reference	Methods	Alteration	Genes	Number of patients	Frequency in BPDCN (%)
Jardin et al,^[33]^ 2011	aCGH/PCR	inactivating mutation	TET2 TP53	13	7/13 (53) 5/13 (38)
Menezes et al,^[34]^ 2014	WES/TS	inactivating mutation	TET2 ASXL1 NRAS NPM1 IKAROS family ZEB2	25	9/25 (36) 8/25 (32) 5/25 (20) 5/25 (20) 5/25 (20) 4/25 (16)
Stenzinger et al,^[23]^ 2014	TS	inactivating mutation; deletion	NRAS ATM MET KRAS IDH2 KIT APC RB1	33	9/33 (27.3) 7/33 (21.2) 3/33 (0.91) 3/33 (0.91) 3/33 (0.91) 3/33 (0.91) 2/33 (0.61) 2/33 (0.61)
Togami et al,^[35]^ 2016	WES/TS	inactivating mutation	ZRSR2 SRSF2 SF3B1 U2AF1 SF3A2 SF3B4	24	7/24 (29) 6/24 (25) 1/24 (4) 1/24 (4) 1/24 (4) 1/24 (4)
Bastidas et al,^[39]^ 2019	WGS/RNA-seq	inactivating mutation; deletion	HNRNPK KZF1 RB1 SYNE1 CDKN1B ETV6 SFRP4	10	8/10 (80) 7/10 (70) 6/10 (60) 5/10 (50) 4/10 (40) 4/10 (40) 3/10 (30)

aCGH = array-based CGH, BPDCN = blastic plasmacytoid dendritic cell neoplasm, PCR = polymerase chain reaction, RNA-seq = RNA sequencing, TS = targeted sequencing, WES = whole-exome sequencing, WGS = whole-genome.

Gene mutations not only help to understand the mechanisms of this rare disease, but also provide targeted strategies for therapeutic intervention. However, how such mutations affect clinical behavior and maintain tumor survival activity remains unclear, and no disease-specific mutated genes have been identified. To date, 3 studies have discovered common features of the heterogeneous genetic background of BPDCN, namely constitutive activation of the NF-κB pathway, the TCF4-/BRD4-dependent transcriptional network, and disruption of cholesterol homeostasis due to the inactivation of liver X receptor (LXR) target genes.^[34,49,50]^

## 7. Traditional treatment: chemotherapy combined with HSCT

Due to the rarity of BPDCN and the difficulty of its diagnosis, most of the clinical information on its treatment comes from retrospective studies. Historically, 3 main conventional chemotherapy regimens are employed for BPDCN: AML regimens such as ICE (idarubicin, cytarabine, etoposide) or MICE (mitoxantrone, cytarabine, etoposide), acute lymphoblastic leukemia (ALL) regimens such as Hyper-CVAD (hyperfractionated cyclophosphamide, vincristine, adriamycin, Adriamycin, and dexamethasone) alternated with methotrexate and cytarabine, and lymphoma regimens such as CHOP (cyclophosphamide, adriamycin, vincristine, prednisone) or CHOP-like regimens (CHOP + etoposide).^[3]^ BPDCN is sensitive to initial chemotherapy, with a complete remission rate of 53% to 89%, and ALL-based chemotherapy regimens are more effective than the other 2 types. However, even so, > 60% of patients experience irreversible relapses and eventually die from progressive disease, with a median survival of 12 to 18 months.^[3,4]^ Studies have shown that consolidation therapy with HSCT [allogeneic (allo) or autologous] after the first complete remission (CR1) significantly improves overall patient survival, and allo-HSCT has a higher remission rate and a lower relapse rate than auto-HSCT.^[5–7]^ Therefore, eligible patients should receive allo-HSCT as soon as possible after CR1. In fact, the vast majority of patients with BPDCN are frail and elderly and exhibit difficulty in tolerating intense chemotherapy. In addition, factors such as limited donor availability have limited the widespread clinical dissemination of chemotherapy combined with HSCT.

## 8. Targeted therapy: CD123

Recently, the introduction of targeted therapies has provided hope for the treatment of BPDCN (Table 2). CD123 (interleukin-3 receptor α chain) is a membrane protein that is overexpressed on the cell surface of various hematological malignancies, including AML, ALL, and BPDCN. However, CD123 is rarely or not expressed on normal hematopoietic stem cells and mature hematopoietic cells, and this significant difference in expression has begun a new era of CD123-targeted therapy. Common CD123-specific targeted therapies include recombinant fusion proteins, chimeric antigen receptor (CAR) T cells, antibody–drug conjugates, and bispecific antibodies.

**Table 2:** Common targeted therapy agents for BPDCN.

Target	Agent	Mechanism	Clinical Trials
CD123	Tagraxofusp	CD123-mediated DT internalization inhibits protein synthesis	NCT00397579 (Approved)
	Anti-CD123 CAR T-cell	Antigen-specific T-cell-mediated cytotoxicity	NCT04109482 NCT03203369
	IMGN632	CD123 antibody conjugated to DNA alkylating agent	NCT03386513
CD123 and CD3	XmAb14045	Bispecific antibody-mediated cytotoxicity	NCT02730312
BCL-2	Venetoclax	Apoptosis via BCL-2 inhibition	NCT03485547
Epigenetic aberrations	5-Aza/Decitabine	Hypomethylating agent	NCT03113643
NF-κB signaling	Bortezomib	Blocks the degradation of ubiquitinated IκB	/
TCF4-/BRD4-network	BET inhibitions	Disruption of TCF4-/BRD4-network	/
LXR	LXR agonists	Restores cholesterol homeostasis and induces apoptosis	/
Multiple (angiogenesis,inflammation)	Lenalidomide	Reduction of pro-inflammatory cytokines like TNF-α, IL-1, and IL-6	/

BET = bromodomain and extra-terminal, BPDCN = blastic plasmacytoid dendritic cell neoplasm, LXR = liver X receptor.

### 8.1. Recombinant fusion protein therapy: tagraxofusp

Tagraxofusp (SL-401) is the most promising targeted agent for the treatment of R/R BPDCN and the first novel drug approved by the U.S. Food and Drug Administration (FDA) in 2018 for patients aged 2 years and older with BPDCN. Tagraxofusp is a recombinant protein consisting of truncated diphtheria toxin fused to human interleukin-3 (IL-3). It specifically binds to the natural receptor (IL-3R/CD123) overexpressed by BPDCN through the IL-3 structural domain, allowing diphtheria toxin to enter the cytosol of tumor cells, thereby inhibiting protein synthesis and ultimately inducing apoptosis.^[51,52]^ In the first phase I/II prospective trial for BPDCN, 11 patients received a single course of treatment with SL-401, and 3 patients relapsed and received a second course of treatment. Of the final 9 evaluable patients with BPDCN, 7 (78%) achieved significant responses, with complete and partial remission rates of 56% (5/9) and 22% (2/9), respectively, and a median remission period of 5 months.^[53]^ Although the sample size was small, this study demonstrated the effectiveness of SL-401 as a targeted agent for the treatment of BPDCN and set the stage for a recent multicenter, multicohort phase III clinical trial, which included 32 first-time treated and 15 previously treated (treatment regimens unknown) patients with BPDCN. A total of 29 first-time treated patients received 12 mg/kg/day of tagraxofusp and were included in the efficacy analysis. The final results showed an overall efficiency of 90% in the first-time treated group, with survival rates of 59% and 52% at 18 and 24 months, respectively. The remission rate in the previously treated group was 67%, with a median overall survival of 8.5 months. The most common and most serious adverse reactions were elevated transaminases (64%) and capillary leakage syndrome (19%), respectively.^[54]^ These findings provide ample evidence that tagraxofusp is feasible and effective in the treatment of BPDCN. As a result, this drug is now an important component of BPDCN treatment.

### 8.2. CAR T cell therapy

Anti-CD123 CAR T cells have potential therapeutic value for BPDCN. The preclinical activity of anti-CD123 CAR T cells has been demonstrated in BPDCN, where it selectively kills tumor cells mainly through antigen-specific T cell-mediated cytotoxicity.^[55–57]^ Bole-Richard et al^[58]^ demonstrated that third-generation CD28/4-1BB CD123 CAR T cells can significantly exert anti-BPDCN efficacy by effectively reducing BPDCN blast burden in vivo and eliminating autologous BPDCN blasts in vitro. In addition, they found that these cells exhibit low or no cytotoxicity against normal cells with low CD123 expression in humanized mouse models, indicating potential low-targeting/nontumor toxic effects. Recently, Chahine et al^[59]^ showed that UCART123, composed of allogeneic T cells expressing anti-CD123 CAR and edited by TALEN® nuclease, has potent antitumor activity. In an in vitro experiment, UCART123 cells selectively killed CD123-positive primary BPDCN samples without damaging normal hematopoietic stem cells. Additionally, UCART123 effectively prolonged survival in patient-derived BPDCN xenograft mouse models by reducing the circulating tumor burden. This UCART123 therapy overcomes the limitations of autologous CAR T cell therapy and greatly reduces the risk of graft-versus-host disease. In summary, these studies have laid the theoretical foundation for the treatment of BPDCN with anti-CD123 CAR T cells. On this basis, 2 phase I clinical trials (NCT04109482 and NCT03203369) are underway to investigate the efficacy of these effector CAR T cells in the treatment of BPDCN.

### 8.3. Antibody–drug conjugate: IMGN632

IMGN632 is a novel antibody–drug conjugate that combines its anti-CD123 monoclonal antibody, G4723A, with a DNA monoalkylation payload of indole-benzodiazepine cytotoxic compounds. After IMGN632 binds to CD123^+^ cells, it can be internalized and release the DNA alkylating agent, FGN849, which leads to cell lysis and apoptosis. Moreover, IMGN632 has shown potent antitumor activity in preclinical models of AML, BPDCN, and ALL.^[60–62]^ A phase I clinical trial evaluating the antitumor activity of IMGN632 in patients with R/R AML and other CD123-positive hematologic diseases, including BPDCN, is ongoing (NCT03386513). To date, 12 patients have received IMGN632 (at doses of 0.015–0.18 mg/kg), among whom 4 achieved an objective response and had no significant dose-limiting toxicities. Thus, IMGN632 may become a reasonable candidate for new therapies for BPDCN.

### 8.4. Bispecific antibody: XmAb14045

XmAb14045 is a bispecific monoclonal antibody targeting both CD3 and CD123.

It can bind CD3^+^ T cells tightly to CD123^+^ cells and then specifically kill tumor cells through T cell-mediated cytotoxicity.^[63]^ A clinical trial is actively enrolling patients with CD123^+^ hematologic malignancies to evaluate the maximum tolerated dose, safety, and preliminary antileukemic activity of XmAb14045 (NCT02730312). While the outcomes of XmAb14045 in patients with BPDCN have not yet been reported, Ravandi et al^[64]^ published preliminary results of XmAb14045 in R/R AML patients and showed that XmAb14045, when administered at doses of 1.3 and 2.3 µg/kg/wk, exerted antileukemic activity with a CR rate of 23%. However, 77% of patients experienced adverse effects in the form of cytokine release syndrome within 1 to 4 hours of dosing, which requires careful management and prevention. In summary, XmAb14045 showed encouraging results in patients with AML, and it may also play an effective role in BPDCN.

## 9. Targeted therapy

### 9.1. BCL-2

Constitutive activation of the NF-κB signaling pathway within BPDCN leads to overexpression of BCL-2.^[49]^ Venetoclax is a selective BCL-2 inhibitor that has demonstrated clinical activity in a variety of hematologic malignancies, including chronic lymphocytic leukemia, myelodysplastic syndromes, and AML.^[45]^ Recently, Montero et al^[65]^ demonstrated in a BPDCN cell line with a BPDCN patient-derived xenograft mouse model that BPDCN is highly dependent on the anti-apoptotic protein BCL-2 and significantly sensitive to venetoclax, which effectively increases mouse survival. In addition, they described the disease response in 2 R/R patients with BPDCN after receiving venetoclax. One patient who received 400 mg/day of venetoclax monotherapy had rapid tumor regression and significant lymph node shrinkage, while the bone marrow showed no significant response. Another patient treated with the same dose of venetoclax showed significant improvement in skin lesions and multiple lymph node lesions, and a 41% reduction in bone marrow BPDCN blastocyte count.

Various case reports have confirmed that the application of venetoclax in patients with overexpression of BCL-2 results in a modest prolongation of overall survival.^[66–68]^ However, relapse is common, and monotherapy with venetoclax does not appear to be sufficient for most R/R patients with BPDCN to achieve long-term disease-free survival. However, DiNardo et al^[69]^ found a synergistic effect of venetoclax in combination with hypomethylating agents to control disease progression and achieve persistent CR. Additionally, a case series reported that 10 patients with R/R BPDCN showed significant disease response to combination treatment with hypomethylating agents + venetoclax, and 2 of them successfully completed allo-HSCT after CR.^[70]^ Overall, the clinical efficacy of this combination regimen has been preliminarily validated, and it could serve as the bridge to HSCT. A clinical trial is currently evaluating the safety of venetoclax in combination with other drugs for BPDCN (NCT03485547).

### 9.2. Epigenetic aberrations

Mutations in genes involved in DNA methylation pathways (e.g., TET2, DNMT3A, IDH1, IDH2) are usually associated with poor prognosis of BPDCN. Moreover, 5-azacytidine (5-Aza) and decitabine are 2 common hypomethylating agents that have been approved by the FDA to treat myelodysplastic syndromes. After whole exome sequencing of 14 patients with BPDCN, Sapienza et al^[71]^ identified epigenetic dysregulation as a potential therapeutic target for BPDCN. They demonstrated that the combination of 5-Aza and decitabine controlled disease progression in a preclinical mouse model of BPDCN. Studies have shown that 5-Aza monotherapy exhibits some clinical efficacy in treating BPDCN, but does not achieve long-term CR.^[72,73]^ Togami et al^[74]^ found that BPDCN resistance to tagraxofusp is associated with downregulation of DPH1, a key regulator of intracellular target delivery of diphtheria toxin, and that 5-Aza can restore DPH1 expression and tagraxofusp sensitivity. Furthermore, they successfully demonstrated that in a BPDCN patient-derived xenograft model, combination treatment with tagraxofusp and 5-Aza significantly improved the overall survival of mice. Therefore, the authors conducted a phase I clinical trial to evaluate the efficacy of tagraxofusp in combination with 5-Aza in the treatment of myeloid malignancies (NCT03113643). Moreover, a recent preclinical study found that 5-Aza can increase the cytotoxicity of anti-CD123 CAR T cells to AML without causing inflammatory organ damage or hematopoietic insufficiency, providing a theoretical basis for subsequent clinical trials combining the 2 to treat AML.^[75]^ In conclusion, combining hypomethylating agents with other drugs for the treatment of myeloid malignancies exhibits potential and is expected to provide a new treatment modality for BPDCN.

## 10. Potential therapeutic targets

### 10.1. NF-κB inhibitors

NF-κB is a key signaling pathway in BPDCN, and targeting this pathway may serve as a promising therapeutic approach for BPDCN. Bortezomib, a first-generation proteasome inhibitor that effectively inhibits the activation of the NF-κB pathway, has been approved by the FDA for a variety of hematological malignancies, including multiple myeloma and mantle cell lymphoma.^[76]^ Sapienza et al^[49]^ found that BPDCN showed constitutive activation of the NF-κB pathway, and treatment of the BPDCN cell line, CAL-1, with bortezomib inhibited cell proliferation and induced significant cytotoxic responses. Subsequently, Philippe et al^[77]^ further demonstrated that bortezomib effectively inhibited the phosphorylation of the NF-κB subunit, RelA in BPDCN cells both in vivo and in vitro, thereby significantly improving the survival rate of mice. Furthermore, they found that the cytotoxic effect of bortezomib was significantly enhanced when combined with classical chemotherapeutic agents. Recently, Economides et al^[78]^ reported that a patient with refractory BPDCN achieved CR after receiving 2 cycles of bortezomib in combination with lenalidomide, an anti-inflammatory and anti-angiogenic immunomodulator capable of showing preclinical activity in a mouse model of BPDCN xenograft.^[79]^

These clinical and preclinical findings provide a theoretical basis for the use of bortezomib in BPDCN. Prospective clinical trials are warranted to confirm these findings.

### 10.2. BRD4 inhibitors

The survival of BPDCN is highly dependent on TCF4- and BRD4- transcription networks. E-box transcription factor TCF4 regulates a series of target genes related to BPDCN (e.g., MYC, BCL-2), and its downregulation leads to the loss of BPDCN-specific gene expression programs, thus causing cell apoptosis.^[34]^ In recent years, the development of BRD4 inhibitors has challenged the traditional belief that transcription factors cannot be targeted in a clinical setting.^[80]^ BRD4 is a member of the bromodomain and extra-terminal family, and the activity of TCF4 is dependent on BRD4. High-throughput drug screening has shown that BRD4 inhibitors can downregulate TCF4 and disrupt the BPDCN-specific transcription network controlled by TCF4-dependent super enhancers, leading to the apoptosis of BPDCN cells.^[34]^ Emadali et al^[29]^ found that the BRD4 inhibitor, JQ1 can reduce the overexpression of the long-chain non-coding RNA gene (linc-RNA-3q) in BPDCN cells, which is associated with the programmed regulation and G1/S transition of leukemia stem cells. Inhibition of linc-RNA-3q expression can block the BPDCN cell cycle and reduce cell line replication. In addition, in an in vivo xenotransplantation model of BPDCN, continuous treatment with JQ1 significantly inhibited the growth of the BPDCN cell line, CAL-1. In conclusion, these data suggest that BRD4 inhibitors may have therapeutic effects on BPDCN.

### 10.3. LXR agonists

Cholesterol plays a key role in cell growth and proliferation, and cholesterol homeostasis is strictly controlled by the LXR signaling pathway.^[50]^ In 2016, Ceroi et al^[50]^ found that the downregulation of LXR target genes related to cholesterol homeostasis in BPDCN caused excessive accumulation of cholesterol in leukemia cells, resulting in the highly proliferative characteristics of BPDCN. Then, the primary BPDCN cells and BPDCN cell lines were treated with LXR agonists, which could restore the expression of LXR target genes, increase cholesterol efflux, inhibit cell proliferation, and induce cell death. In addition, in an in vivo xenotransplantation model, LXR agonist treatment effectively reduced the degree of infiltration of BPDCN cells and significantly improved the survival rate of mice. In conclusion, LXR agonists are expected to provide a new treatment option for BPDCN.

## 11. Future directions

BPDCN is a clinically rare and aggressive hematologic malignancy with an extremely poor prognosis. The molecular landscape of BPDCN should be further explored in the future. A better understanding of the genetic and molecular biological alterations of the disease could lay the theoretical foundation for the design of new antitumor drugs. With the improved understanding of the biological properties of BPDCN and the development of new technologies, future research hotspots are mainly focused on targeted drug development and combination drug applications, including anti-CD123-specific drugs, BCL-2 inhibitors, and demethylation drugs. Tagraxofusp, as the first targeted drug approved for BPDCN with controlled toxicity and a lasting response, has been proven to be effective. However, for eligible patients, whether tagraxofusp can be an effective treatment to avoid transplantation requires further study. The usefulness of other potential targeted agents, such as BRD4 inhibitors, LXR agonists, and NF-κB inhibitors has not been confirmed in prospective clinical trials, despite having shown preclinical activity, and further large-scale prospective studies are needed to confirm these findings in the future.

## 12. Conclusions

Although the understanding of the etiology and pathophysiology of BPDCN is growing, many issues remain unresolved. Research has uncovered the cytogenetics and molecular biology of the pathogenesis of BPDCN, though neither of them is specific. Additionally, commonality in the heterogeneous genetic background of BPDCN should be further explored. Although the activation of the NF-κB pathway, the disruption of cholesterol balance, and the destruction of the TCF4-/BRD4-dependent transcription network have been found in BPDCN, the above changes do not fully reveal the regulatory mechanisms involved in BPDCN development. The changes in the gene expression profile at the molecular level should be further studied to reveal the pathogenesis.

**Figure 1.**
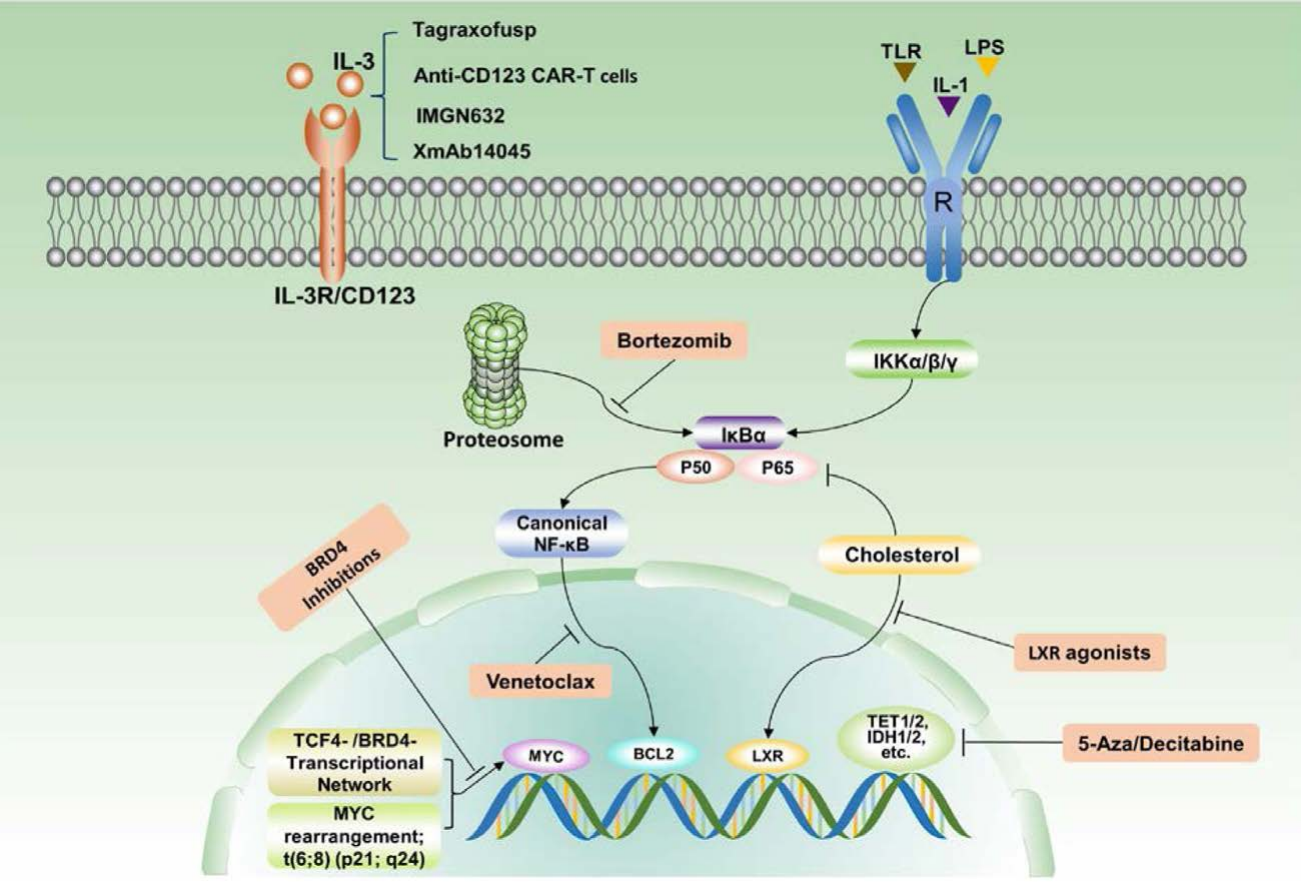
Potential biomarkers of BPDCN and the latest therapeutic approaches. BPDCN = blastic plasmacytoid dendritic cell neoplasm.

In clinical practice, chemotherapy combined with HSCT remains the most important treatment strategy for young patients and elderly patients who meet the transplant conditions. After CR1 from ALL chemotherapy, consolidation treatment with allo-HSCT can significantly improve the overall survival of patients. Targeted therapy is a feasible method for the elderly with poor health and some patients with R/R BPDCN (Fig. 1). At present, tagraxofusp, a targeted drug for CD123, has been approved by the FDA for BPDCN, and other specific drugs targeting CD123 are undergoing clinical trials. In addition, venetoclax and hypomethylating agents have shown clinical activity, and clinical trials of their combination for BPDCN are being actively carried out. These new drugs are expected to improve the curative effect and reduce toxic reactions, but further research is needed to determine their safety. In addition, NF-κB inhibitors, BRD4 inhibitors, and LXR agonists can also be used as potential therapeutic strategies for BPDCN. Gene development and targeted therapy should be combined to explore more effective treatment methods for BPDCN.

## Author contributions

**Conceptualization:** Yemin Wang, Lv Zhou.

**Data curation:** Li Xiao, Lili Yin, Yanjuan Deng.

**Funding acquisition:** Huan Deng.

**Methodology:** Yemin Wang.

**Software:** Lili Yin, Lv Zhou, Yanjuan Deng.

**Supervision:** Huan Deng.

**Writing – original draft:** Yemin Wang, Li Xiao.

**Writing – review & editing:** Huan Deng.
